# Presence of globally prominent multidrug-resistant genotypes of Salmonella enterica serovar Typhi in New York State, 2016–2023

**DOI:** 10.1099/mgen.0.001816

**Published:** 2026-09-02

**Authors:** Samantha K. Lindberg, Ana Beatriz Garcez Buiatte, Odion O. Ikhimiukor, Leticia Roberta Martins Costa, Samantha E. Wirth, Kimberlee A. Musser, Lisa A. Mingle, Cheryl P. Andam

**Affiliations:** 1 Department of Biological Sciences, University at Albany, State University of New York, Albany, NY, USA; 2 Wadsworth Center, New York State Department of Health, Albany, NY, USA

**Keywords:** antimicrobial resistance, genomics, genotypes, *Salmonella enterica*, serovar Typhi

## Abstract

The human-restricted enteric pathogen *Salmonella enterica* serovar Typhi (*S*. Typhi) is the causative agent of the life-threatening typhoid fever. Although *S*. Typhi incidence is relatively low in the USA, routine surveillance of *S*. Typhi is critical to track the emergence and spread of high-risk lineages in non-endemic areas. In this study, we analysed 151 genomes of *S*. Typhi isolates from patients who were clinically confirmed with typhoid fever across New York State between 2016 and 2023. We used the GenoTyphi classification scheme and identified established multidrug-resistant and extensively drug-resistant lineages. We detected the presence of the globally widespread genotype 4.3.1 (haplotype 58) and its derivative 4.3.1.1.P1, which recently emerged in Pakistan, as well as the Bangladesh-restricted lineages 3.3.2.Bd1 and 3.3.2.Bd2 in our dataset. Ten mutations and 14 acquired genes associated with antimicrobial resistance (AMR) were present across the entire population, with 86.8% of the genomes possessing at least one of these AMR determinants. The *gyrA* S83F mutation conferring quinolone and triclosan resistance was the most frequently detected (94 genomes). Combinations of *dfrA7+catA1* (resistance to trimethoprim and chloramphenicol, respectively) and *sul2+aph(3″)-Ib+aph(6)-Id* (resistance to sulphonamide and aminoglycosides, respectively) co-occurred frequently and were associated with IncQ and IncY plasmid replicons. Phylogenetic contextualization against a global dataset of 1,643 genomes from 20 countries across five continents, including other parts of the USA, from the same time period showed geographic intermingling, suggesting the spread of high-risk genotypes of international origins to New York State. Altogether, these findings reveal the presence of globally dominant resistant genotypes that are likely facilitated by human travel in New York State, where typhoid fever is not endemic. Long-term genomic surveillance is critical to AMR profiling, identifying genotypic shifts in regional *S*. Typhi populations, monitoring transmission routes and guiding effective public health interventions.

**Impact Statement**

The public health and clinical burden of *Salmonella enterica* serovar Typhi (*S*. Typhi) infections remain distressingly high, exacerbated by the rapid spread of antimicrobial-resistant genotypes traversing countries and continents. Our genomic analysis of *S*. Typhi in New York State reveals the presence of globally dominant genotypes carrying multiple antimicrobial resistance determinants. These findings expand existing knowledge about the diversity and genomic features of *S*. Typhi in non-endemic regions, such as New York State. These data will be valuable in implementing efforts to minimize secondary transmission and public health control measures against *S*. Typhi that are most effective at local and regional scales.

## Data Summary

Genome sequence data of *S*. Typhi isolates used in this study are available in the NCBI Sequence Read Archive (SRA). SRA, PulseNet and BioSample accession numbers for the New York State *S*. Typhi genomes are listed in Table S1, and accession IDs for the global genomes obtained from TyphiNET are listed in Table S2. Raw output data analysed in this study are included in Tables S3–S10.

## Introduction

*Salmonella enterica *serovar Typhi (*S*. Typhi) is the causal agent of typhoid fever, a life-threatening infection that is transmitted via the faecal–oral route [1]. Typhoid fever is a febrile multisystemic illness characterized by fatigue, abdominal pain, headache, constipation, vomiting and pink rashes [1]. If treatment is delayed or inadequate, manifestations can become progressively severe and include delirium, intestinal bleeding, intestinal perforation, meningitis, sepsis and hepatosplenomegaly (simultaneous enlargement of both the liver and the spleen) [1]. In contrast to non-typhoidal serovars (e.g*.* Enteritidis, Typhimurium, Newport and Infantis) that are host generalists and typically cause self-limiting gastroenteritis, *S*. Typhi is a strictly human pathogen [2, 3].

The global burden of disease caused by typhoid fever remains distressingly high. Worldwide, an estimated 9.2 million cases of typhoid fever and 110,000 deaths occurred in 2019 based on modelling data from the Global Burden of Disease study [4]. From 2017 to 2022, seven confirmed large-scale outbreaks of typhoid fever were identified in four countries (the Philippines, Zimbabwe, China and Pakistan) by the Centers for Disease Control and Prevention’s (CDC) Global Disease Detection Operation Center [4]. In a meta-analysis of global typhoid incidence from 2018 to 2024, incidence estimates were highest in Africa and South Asia and among children 2 to <5 years old [5]. *S*. Typhi remains endemic in low- to middle-income countries that have limited access to clean water, adequate sanitation and hygiene (known collectively as WASH) [5, 6]. Moreover, individuals may become carriers of *S*. Typhi and continue to shed bacteria for months up to years after recovering from acute infection or without ever developing clinical symptoms [7, 8]. Asymptomatic, convalescent and post-symptomatic shedding creates a persistent reservoir that facilitates sustained transmission via the faecal–oral route [7]. Improvements in water and sanitation infrastructure as well as the widespread use of typhoid conjugate vaccines [9] are therefore critical in preventing and controlling typhoid fever.

Typhoid fever requires prompt treatment with appropriate antimicrobial agents. However, the effectiveness of antimicrobial therapy against *S*. Typhi has been threatened by the emergence and spread of antimicrobial resistance (AMR) [10, 11]. From 2010 to 2018, ~35% of reported *S*. Typhi isolates in Asia and 75% of isolates in Africa exhibited resistance against frontline antibiotics historically used against *S*. Typhi infections, i.e. chloramphenicol, ampicillin and trimethoprim–sulfamethoxazole [10]. Concurrent resistance to all three antibiotics defines multidrug-resistant (MDR) *S*. Typhi. A recent modelling study by the GRAM Typhoid Collaborators estimated that the prevalence of MDR *S*. Typhi infections in sub-Saharan Africa rose sharply from 6.0% in 1990 to 72.7% in 2019 [12]. In 2016, Pakistan was the first country to report extensively drug-resistant (XDR) *S*. Typhi, characterized by MDR plus additional resistance to fluoroquinolones and third-generation cephalosporins, facilitated by an IncY plasmid harbouring *qnrS* and *bla*_CTX-M-15_ genes [13]. Pakistan remains the epicentre of XDR *S*. Typhi, but travel-related XDR cases have been increasingly documented in other countries [14, 15]. MDR and XDR *S*. Typhi infections are consistently associated with limited treatment options, severe clinical manifestations, adverse complications and prolonged hospitalization, thus posing a serious threat to patient outcomes [16–18].

International collaboration in whole-genome sequencing of clinical *S*. Typhi isolates has led to the development of GenoTyphi, a lineage genotyping and nomenclature scheme specific for *S*. Typhi [19, 20]. GenoTyphi uses marker single nucleotide variants to assign *S*. Typhi genomes to phylogenetic clades and subclades to systematically track the emergence and dissemination of high-risk resistant lineages, as well as facilitate epidemiological investigations including travel-associated incidence or local outbreaks [19, 20]. For example, the MDR haplotype 58 (H58), classified as genotype 4.3.1 under the GenoTyphi scheme, emerged in India in the late 1980s and is now globally distributed [21]. Over the past two decades, H58/4.3.1 has evolved into diverse sublineages with unique genetic features and acquired AMR determinants in various parts of Asia and Africa [22–25]. Nonetheless, increasing reports of non-H58 genotypes with distinct resistance patterns from different parts of the world pose new challenges to controlling typhoid fever [11, 26, 27].

In high-income non-typhoid-endemic countries such as the USA, typhoid fever is a reportable disease, and cases are often associated with international travel, contact with a traveller from a country where typhoid fever is endemic or exposure to food items handled by a chronic carrier [28–30]. Here, we aim to characterize the *S*. Typhi population in New York State, a non-endemic area for typhoid fever, in terms of its pool of AMR determinants and genetic relationships to lineages from other countries. We analysed short-read genome sequences of 151 clinical isolates collected from 2016 to 2023 and contextualized them against a global dataset (*n*=1,643 genomes). Our results reveal the presence of the globally prominent antimicrobial-resistant genotype 4.3.1/H58 and its XDR sublineage 4.3.1.1 .P1. Understanding the population genomic structure of *S*. Typhi in non-endemic areas such as New York State is critical to implementing appropriate and effective public health strategies to minimize the broader dissemination of high-risk resistant genotypes both at local and global scales.

## Methods

### Collection of bacterial isolates

Infections due to *S. enterica*, including *S*. Typhi, are reportable diseases in New York State. Clinical laboratories throughout the state are required to submit all *Salmonella* isolates to the Wadsworth Center, the public health laboratory of the New York State Department of Health, in line with New York State’s 2020 Laboratory Reporting of Communicable Disease guidelines (https://wadsworth.org/sites/default/files/WebDoc/CDRG%20NYState%202020_101920%202.pdf). *Salmonella* surveillance in New York State is part of the national-level FoodNet [31] and PulseNet [32] surveillance networks run by CDC. *S*. Typhi isolates submitted to the Wadsworth Center for confirmatory testing and genome sequencing during the routine course of the state public health surveillance programme for *Salmonella* from 2016 to 2023 were used in this study. Bacterial isolates were submitted by New York State healthcare providers from individuals who had been clinically confirmed with *Salmonella* infection by either the clinical laboratory or the Wadsworth Center. A single bacterial isolate was obtained from each patient. However, New York State does not require healthcare providers to provide patient information, including specific disease symptoms, patient outcomes and epidemiological data (e.g. travel history, social contacts and treatment regimen) to the Wadsworth Center. We did not obtain, use or report patient protected health information in this study. Therefore, informed consent was not required. This work has been determined to be exempt from human subject research by the Institutional Review Board of the Wadsworth Center.

### Identification of bacterial isolates as *S*. Typhi

*Salmonella* species and serotype detection was performed as previously described [33]. Briefly, all isolates received from local laboratories were streaked on an Endo agar plate for purity, and a single colony was transferred to a Brain Heart Infusion (BHI) agar slant and a triple sugar iron (TSI) slant. Isolates with a typical reaction for *S*. Typhi in the TSI slant with a small amount of H_2_S production were sent for conventional serotyping using agglutination tests with known antibodies to confirm *S*. Typhi.

### DNA extraction, DNA library preparation and whole-genome sequencing

We implemented a streamlined protocol for a high-throughput whole-genome sequencing approach that maximizes efficiency and cost-effectiveness in a public health laboratory [34]. Briefly, *S*. Typhi isolates were grown on BHI slants and incubated at 37 °C for 24–48 h. A 1-µl inoculating loop was used to collect bacterial growth from each culture and lysed using a solution of 180 µl buffer Aniset/Animal Tissue Lysis buffer and 20 µl Proteinase K (50 µg µl^−1^) per sample for 60 min on a shaking thermomixer at 56 °C. Genomic DNA was extracted using the standard QIAamp DNA Blood Minikit protocol or QIAamp 96 DNA QIAcube HT kit on the automated QIAcube or QIAcubeHT (Qiagen, Germantown, MD, USA). DNA was quantified using Quant-iT™ dsDNA High-Sensitivity Assay Kit and the Fluoroskan™ Microplate Fluorometer in 96-well format (Thermo Fisher Scientific, Waltham, MA, USA).

Library preparation and sequencing were carried out at Wadsworth Center’s Advanced Genomic Technologies Cluster. DNA libraries were prepared using Illumina Nextera XT or Illumina DNA Flex kits following manufacturer’s protocols. Sequencing was carried out with either an Illumina MiSeq (2×250 chemistry and v2 kits) or an Illumina NextSeq500 (2×150 chemistry and v2 kits). Demultiplexing was done using the Illumina BCL2FASTQ script. Paired-end reads were classified by Kraken v0.10.5-beta [35]. The prebuilt Minikraken 20171019_8 GB database was used for the reference sequence database. The Kraken output served as input in Krona tools to generate the taxonomic assessment (https://github.com/marbl/Krona/wiki). To ensure that minimum quality thresholds established by the Center for Food Safety and Applied Nutrition (CFSAN) were followed, sequence read quality was assessed using MicroRunQC implemented on the GalaxyTrakr platform of Galaxy [36] or thresholds established by PulseNet using Bionumerics v7.6.3. For all genomes sequenced, *de novo* coverage was ≥30×, average quality (*Q*) score was ≥30, and assembly lengths fell within the acceptable range of 4.4–5.7 Mbp for *Salmonella* genomes set by PulseNet. Genome sequences were submitted to the Pathogen Detection database of the National Center for Biotechnology Information (NCBI) (https://www.ncbi.nLm.nih.gov/pathogens/) in real time.

### *De novo* genome assembly, sequence quality check and annotation

*De novo* assembly of contigs were generated from paired-end sequences using the Shovill v1.1.0 pipeline (https://github.com/tseemann/shovill), implementing the SPAdes v3.14.1 assembler and the -trim option to remove adapter sequences [37]. We used CheckM v1.2.2 [38] and QUAST v5.0.2 [39] to assess the quality of assembled genomes. All 151 genomes obtained from the Wadsworth Center had >90% completeness, <5% contamination, <200 contigs and an N50 score of >40,000 bp. All 151 genomes were included in all downstream analyses (Fig. S1 and Table S1, available in the online Supplementary Material).

For the global *S*. Typhi analysis, we retrieved sequences from the TyphiNET database (accessed in July 2025; https://www.typhi.net/) [40] and included those genomes that were collected from 2016 onwards to match the first year of collection of the genomes from New York State. Genomes lacking accession numbers, year and country of isolation and travel information were excluded. To minimize the impact of oversampled countries, 151 genomes were selected at random from countries with a high number of genomes to match the number of the New York State genomes. This was also done because of limitations in computational resources in our institution to analyse the entire global TyphiNET dataset (*n*=11,836 genomes). For countries with <151 genomes, all available genomes were included. The corresponding .sra files were retrieved from the NCBI Sequence Read Archive (SRA) and converted to .fastq format using the NCBI SRA toolkit v3.2.1 (https://github.com/ncbi/sra-tools) and then assembled and checked for quality as described above. Five genomes from Kenya and one genome from Rwanda were excluded from downstream analyses due to a high number of contigs (>200). Altogether, the global dataset comprised 1,643 additional genomes representing 20 countries across 5 continents, including 151 from other parts of the USA (Table S2). Genome annotation was carried out using Bakta v1.4.0 [41]. Sequence quality metrics for the New York State dataset and New York plus global dataset are presented in Tables S1 and S2, respectively.

### Pan-genome analyses and phylogenetic tree reconstruction

We used Panaroo v1.3.3 [42] to characterize the collective set of genes or pan-genome [43] for both datasets. We implemented Panaroo with strict mode with the ‘remove-invalid-genes’ option enabled and ran it separately for the first dataset (New York State genomes only, *n*=151) and the second dataset (New York plus global genomes, *n*=1,794). For each dataset, genes present in ≥95% of the genomes (or core genes) were identified: *n*=4,473 core genes in the first dataset and *n*=4,530 core genes in the second dataset. Results of the Panaroo analysis for the New York dataset and New York plus global dataset are presented in Tables S3 and S4, respectively. A summary of the Panaroo results for the New York State dataset is shown in Fig. S2.

Nucleotide sequence alignment of each gene family was carried out using MAFFT [44] implemented in Panaroo. SNPs in the core gene alignment were extracted using SNP-sites v2.5.1 (https://github.com/sanger-pathogens/snp-sites): 8,197 core SNPs in the first dataset and 37,633 SNPs in the second dataset. We used the alignments of core gene SNPs as input in IQ-TREE v2.1.4-beta [45] to build maximum likelihood phylogenetic trees. The ModelFinder algorithm built into IQ-TREE was employed to determine the best-fit model [46]. The general time-reversible nucleotide substitution model [47] with empirically calculated base frequencies and ascertainment bias correction (GTR+F+ASC) was chosen according to the Bayesian information criterion for both datasets, with three and five categories of rate heterogeneity in the first and second datasets, respectively. Trees were visualized, annotated and rooted at the midpoint using RStudio v4.5.1 [48] implementing ape v5.8.1 [49], ggtree v3.16.0 [50] and phytools v2.4.4 packages [51].

To verify the phylogenetic relationships in this second (global) dataset, we built a phylogenetic tree following the methodology used in the GenoTyphi scheme [20]. Here, the global dataset genomes were mapped to a reference sequence of *S*. Typhi CT18 (GenBank accession: AL513382) using Snippy v4.6.0 (https://github.com/tseemann/snippy) and recombinant regions from the resulting alignment were masked using Gubbins v3.2.1 [52]. The recombination-free alignment was used as input to build a maximum likelihood phylogenetic tree as described above (Fig. S3).

### *In silico* genotyping of *S*. Typhi

We used the GenoTyphi classification scheme v2.1 [19, 20] and Mykrobe v0.13.0 [53] to characterize the genotypes present in the New York State dataset. Genotyping results are presented in Table S5.

### *In silico* detection of AMR determinants and plasmid replicon types

To identify genes and point mutations associated with AMR, we ran AMRFinderPlus v4.0.22 and its accompanying NCBI-compiled database v2025-03-25.1 using thresholds of 60% sequence coverage and 80% sequence identity [54]. In the New York State dataset, coverage ranged from 72.69 to 100%, with nine instances of *sul2* and one copy of *aph(6)-Id* having coverage between 72.69 and 80.07%. All other AMR determinants had ≥97% coverage. All AMR determinants in the New York State dataset had ≥99.43% coverage. Distribution of AMR determinants in the New York dataset and in global dataset is presented in Tables S6 and S7. Predicted MDR and XDR profiles were determined based on previously described combinations of resistance markers [10, 13]: chloramphenicol, ampicillin and trimethoprim-sulfamethoxazole for MDR and all MDR markers plus fluoroquinolone and third-generation cephalosporin for XDR (Table S8).

Co-occurrence of AMR determinants in the New York State genomes was determined using ReGAIN v1.5.0 (https://github.com/ERBringHorvath/regain_CLI) [55]. AMR elements that occur in <5% and >99% of the genomes were excluded to reduce noise in the Bayesian co-occurrence network caused by rare and ubiquitously occurring genes, respectively. A total of 500 bootstrap replicates was implemented to measure support for the network. A 0.5 significance threshold was used to filter out weakly supported gene pairs. For every pair of AMR genes, we calculated four different co-occurrence metrics (relative risk, conditional probability, bidirectional probability score and fold change). AMR pairs with a relative risk value <1, conditional probability value <0.7, bidirectional probability score <1 and fold change value <1 were excluded from the Bayesian networks (Figs S6 and S7) to show only those gene pairs with strong bidirectional associations. Output of the ReGAIN analysis is presented in Table S9.

We used MOB-recon and MOB-typer v3.1.0 in MOB-suite [56] implementing default parameters to identify putative plasmids on contigs based on the presence of the replicon gene (*rep*) that encodes the plasmid replicon initiator protein [57]. Distribution of plasmid replicon types in the New York dataset is presented in Table S10.

### Data visualization and statistical analyses

**Fig. 1.**
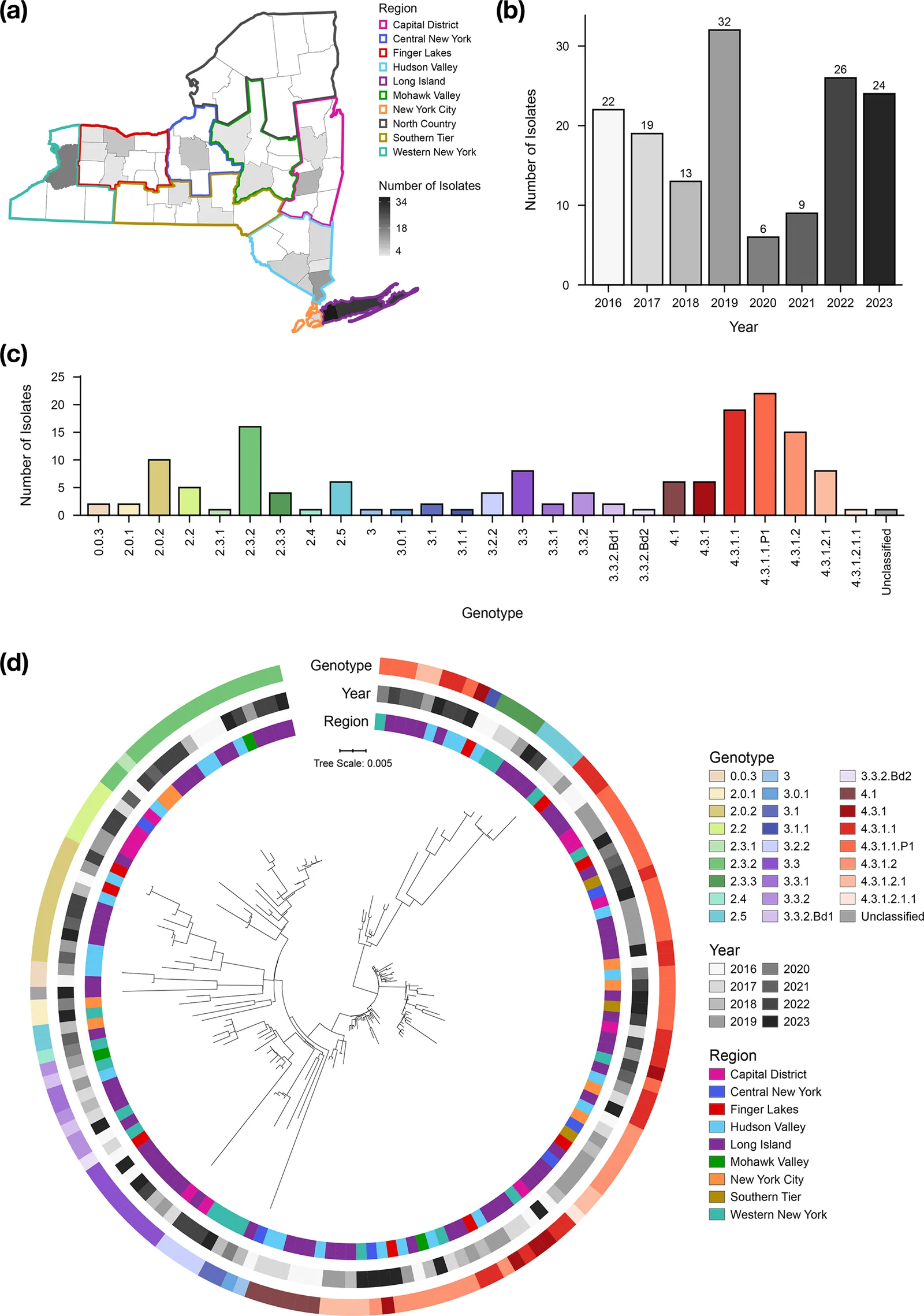
Population structure of *S*. Typhi in New York State from 2016 to 2023 (*n*=151 genomes). (**a**) Map of New York State showing counties with borders coloured according to the major regions in the state. Counties are coloured in different shades of grey according to the number of genomes present. (**b**) Number of *S*. Typhi genomes recovered per year. Data values per year are listed above each coloured bar. (**c**) Number of *S*. Typhi genomes based on the GenoTyphi classification scheme. (**d**) Maximum likelihood phylogenetic tree built using 8,197 SNPs extracted from the 4.77 Mbp sequence alignment of 4,473 core genes. The tree is rooted at the midpoint. Tree scale reflects the number of SNPs/site in the core SNP alignment (8,197 SNPs). Coloured strips around the tree show the geographical region, year of isolation and genotypes (innermost to outermost rings). Detailed information on the genomic features and associated metadata of individual isolates is presented in Table S1.

All figures were generated using RStudio v2025.05.01 [48] and the following packages: ComplexUpset v1.3.3, countrycode v1.6.1, cowplot v1.2.0, ggraph v2.2.1, ggnewscale v0.5.2, ggplot 2 v3.5.2, ggsignif v0.6.4, igraph v2.1.4, patchwork v1.3.1, scales v1.4.0, sf v1.0.21, tidygraph v1.3.1 and tidyverse v2.0.0 [58–71]. The New York State map used in Fig. 1a was retrieved from https://gis.ny.gov/civil-boundaries. The world map was obtained from the R packages rnaturalearth v1.1.0 and rnaturalearthdata v1.0.0 [72, 73].

## Results

### Population genomic structure of *S*. Typhi in New York State

We analysed 151 high-quality genome sequences of *S*. Typhi derived from the *S. enterica* surveillance carried out by the New York State Department of Health (Table S1). Isolates were collected between January 2016 and September 2023. New York State consists of 62 counties grouped into 10 regions. At least 1 *S*. Typhi genome was recovered in 22 counties (Fig. 1a and S4). The highest number of genomes collected were from Long Island, with Nassau and Suffolk counties each reporting 37 and 31 genomes, respectively. The number of genomes per year ranged from 6 (in year 2020) to 32 (in year 2019) (Fig. 1b). However, we note that the variation in the number of genomes across the state is likely a consequence of large differences in human population size, extent of surveillance efforts, resident travel patterns and other socioeconomic activities that differ among counties. Hence, inferences about the *S*. Typhi distribution in New York State should be made only within the context of this current dataset.

The New York State population included 18 diverse genotypes within primary clades 0, 2, 3 and 4 (Fig. 1c, Table S10 and Fig. S4). Nearly half of the genomes (*n*=71, 47.0%) belonged to genotype 4.3.1/H58 or its associated sublineages. The most common genotype in our dataset was 4.3.1.1.P1 (*n*=22 genomes), a 4.3.1 sublineage currently endemic to Pakistan [13, 20]. We also detected the presence of the Bangladesh-associated genotypes 3.3.2.Bd1 (*n*=2 genomes) and 3.3.2.Bd2 (*n*=1 genome) as well as genotype 2.5 (*n*=6 genomes), which is one of two dominant endemic genotypes in Colombia [74]. Phylogenetic analysis of the New York State genomes revealed a lack of geographical or temporal structuring (Fig. 1d).

### *S*. Typhi genomes in New York State carry numerous AMR determinants

**Fig. 2.**
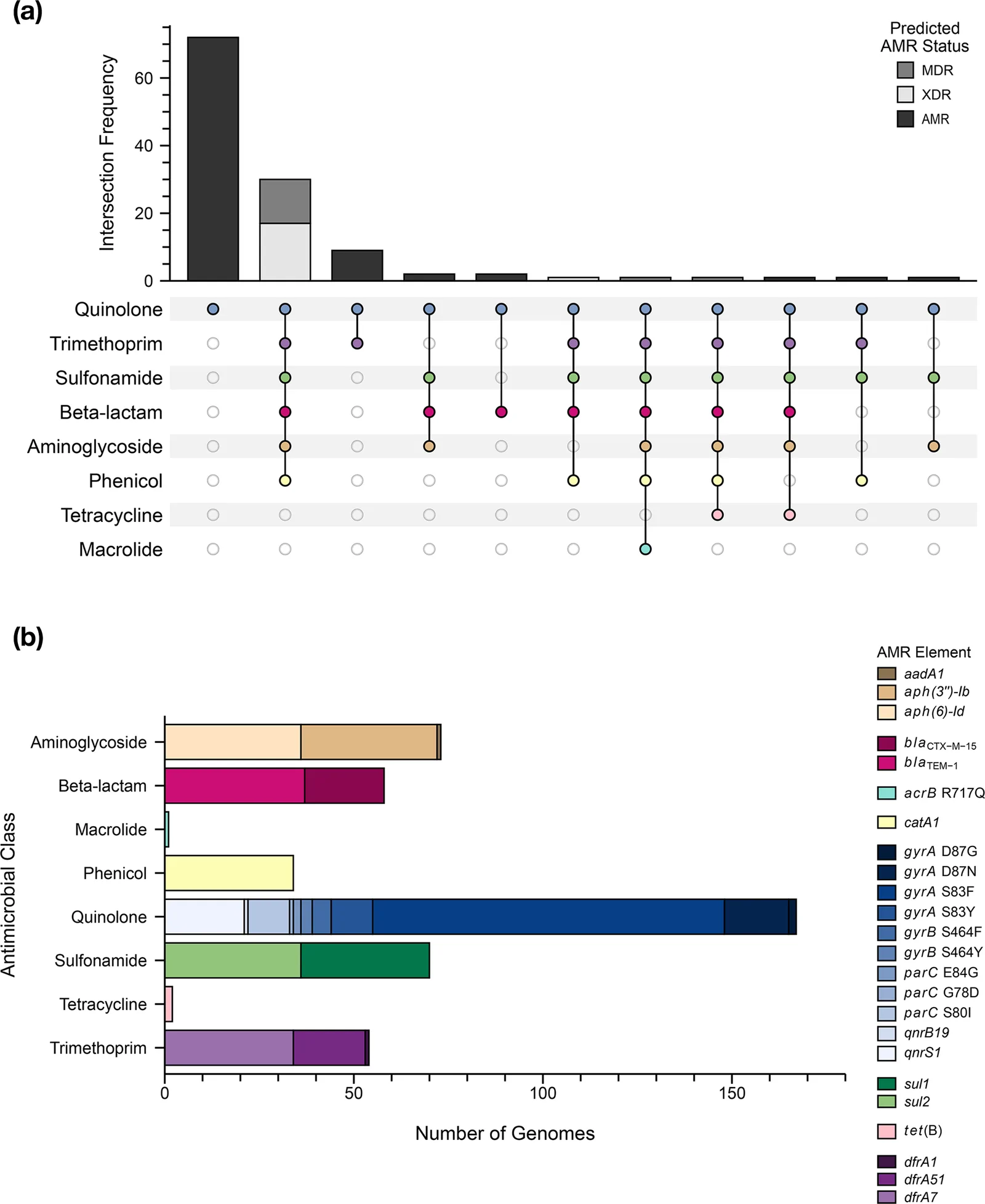
Genetic determinants of AMR in *S*. Typhi genomes from New York State. (**a**) Number of genomes with distinct AMR profiles based on the presence of at least one AMR determinant for each antimicrobial class. The bars are coloured according to predicted AMR status (MDR, XDR) based on known combination of specific resistance markers [10, 13]. Filled dots indicate the presence of an AMR determinant detected for a specific antimicrobial class. (**b**) Number of genomes carrying individual genetic determinants of AMR. The AMR genes in the colour legend are grouped according to antimicrobial class.

We sought to determine the presence of genes and genetic mutations associated with AMR using *in silico* screening of the genomes. A total of 121/151, or 80.1%, of the genomes harboured at least one acquired gene or mutation associated with AMR, with 34 genomes possessing ≥5 AMR determinants (Fig. S5). The entire set of AMR determinants identified in our dataset represented nine classes of antimicrobial agents and 14 unique combinations of AMR determinants (or profiles) (Figs 2a, S5 and S6). Resistance to quinolones was commonly detected in the New York State population (*n*=121 genomes). The diverse AMR profiles detected in our dataset comprised 14 acquired genes and 10 genetic mutations (Fig. 2b) and were differentially distributed among the 151 genomes (Table S6 and Fig. S6). The most variable group of AMR determinants was those that confer quinolone resistance (four *gyrA* mutations, three *parC* mutations and two *qnr* genes). The most common AMR determinant was the S83F substitution in the quinolone resistance-determining region (QRDR) of the DNA gyrase subunit gene *gyrA* (*n*=93 genomes, 61.6% of the dataset). The *gyrA* S83F mutation confers reduced susceptibility to fluoroquinolones and triclosan [75, 76] and has been identified in *Salmonella* isolates worldwide [77]. There were 36 genomes (23.8%) carrying >1 quinolone or quinolone+triclosan resistance determinants. The Bangladesh-associated genotypes 3.3.2.Bd1 and 3.3.2.Bd2 are associated with different mutations in the QRDR of the *gyrA* gene [26], and in our dataset, the two 3.3.2.Bd1 genomes possessed S83F mutations and the one 3.3.2.Bd2 genome harboured a D87N mutation. The Colombian genotype 2.5 that we detected in New York State is also reported to be linked to QRDR mutations [74].

Other frequently detected AMR determinants were *bla*_TEM−1_ (*n*=37; conferring beta-lactam resistance), *aph*(3″)-Ib and *aph*(6)-Id (each present in 36 genomes; aminoglycoside resistance), *sul1* and *sul2* (present in 34 and 36 genomes, respectively; sulphonamide resistance), *dfrA7* (present in 34 genomes; trimethoprim resistance) and *catA1* (present in 34 genomes; phenicol resistance) (Fig. 2b). Classification of *S*. Typhi into MDR and XDR strains has been previously defined [10, 13]. A total of 15 genomes were predicted to be MDR based on the combined presence of ampicillin, chloramphenicol and trimethoprim–sulfamethoxazole resistance markers (*bla*, *catA1* and *dfrA+sul*, respectively), while 18 genomes were predicted to be XDR based on the presence of the MDR markers plus genetic determinants for ciprofloxacin resistance (one QRDR mutation+*qnrS1*) and ceftriaxone resistance (*bla*_CTX-M-15_) (Table S8). In New York State, genotypes 4.3.1.1 (*n*=14 genomes) and 4.3.1.1.P1 (*n*=1) were classified as MDR. A total of 18 genomes belonging to genotype 4.3.1.1.P1 were classified as XDR.

### Co-occurring AMR genes are associated with plasmid replicons

**Fig. 3.**
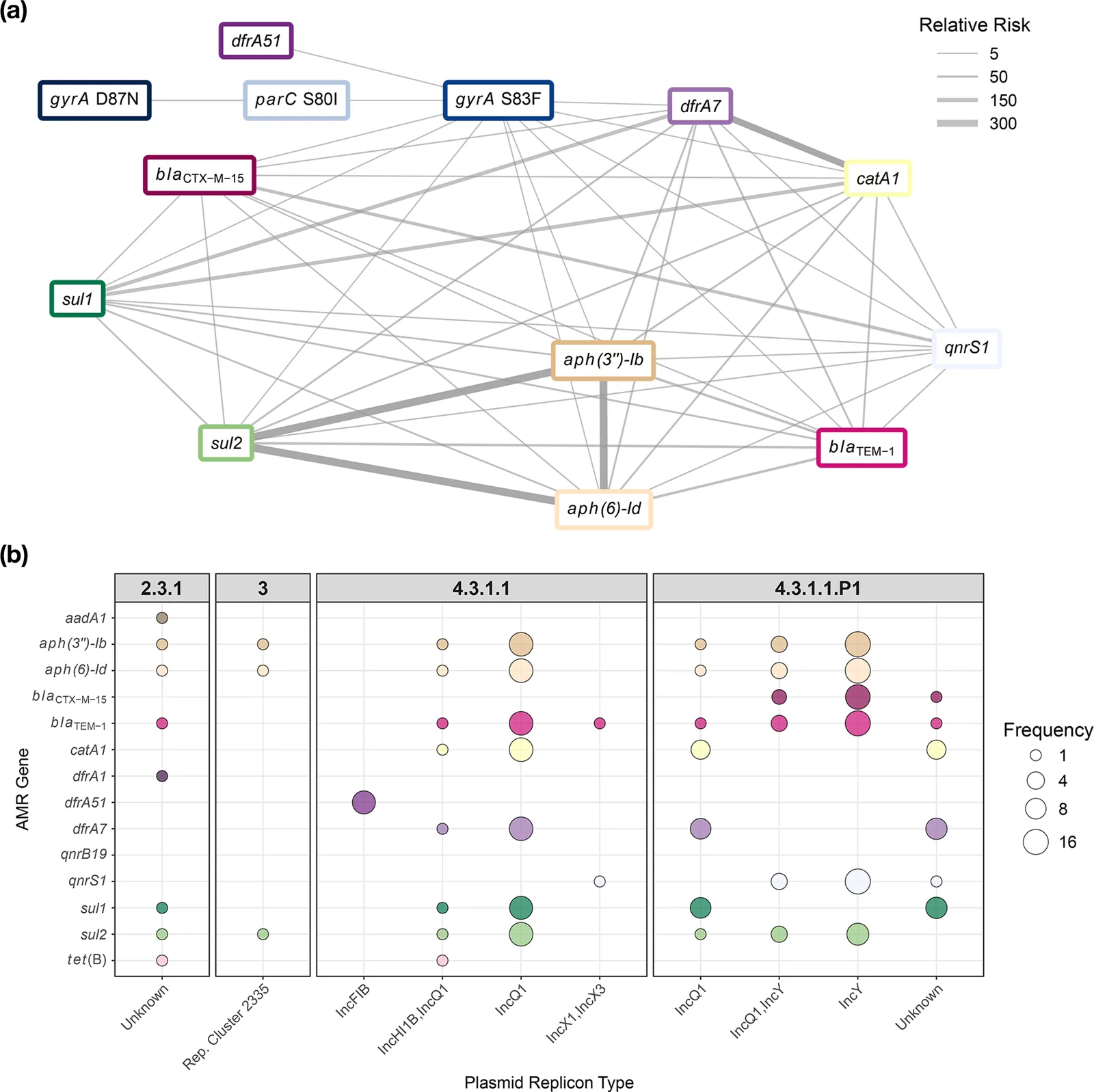
Co-occurrence of AMR determinants. (**a**) Association network showing the co-occurrence of specific AMR determinants based on relative risk. Edge width is scaled by relative risk, which represents the ratio of the probability of observing gene A in the presence of gene B to the probability of observing gene A in the absence of gene B [55]. Nodes represent AMR determinants and are coloured by antimicrobial class. Details of the co-occurrence analysis are presented in Table S10. (**b**) Association of AMR genes with plasmid replicons categorized by genotype. Bubble size indicates the number of genomes with a contig associated with a plasmid replicon and the corresponding AMR genes present.

A total of 13 AMR determinants exhibited strong pairwise co-occurrence, with relative risk values >1, indicating that it is more likely to observe gene A in the presence of gene B (Figs 3a and S7, Table S10). Three QRDR mutations (*gyrA* S83F, *gyrA* D87N and *parC* S80I) and ten resistance genes [*aph*(3″)-Ib, *aph*(6)-Id, *bla*_CTX−M−15_, *bla*_TEM-1_, *catA1*, *dfrA51*, *dfrA7*, *qnrS1, sul1* and *sul2*] associated with resistance to six antimicrobial classes formed a highly interconnected resistance network. Five resistance determinants (*bla*_TEM-1_, *bla*_CTX−M−15_, *gyrA* S83F, *catA1* and *sul2*) had the highest number of linked AMR determinants (Fig. S8). Notably, the strongest co-occurrence was observed between the trimethoprim resistance gene *dfrA7* and the chloramphenicol resistance gene *catA1*, with both genes present together in 34 genomes. We also observed a strong three-way association between the sulphonamide resistance gene *sul2* and the aminoglycoside resistance genes *aph(3″)-Ib* and *aph(6)-Id*; all three genes were present simultaneously in 36 genomes. Accordingly, 32 genomes containing both the *dfrA7–catA1* pair and the *sul2–aph(3″)-Ib–aph(6)-Id* triad were classified as MDR/XDR, corresponding to genotypes 4.3.1.1 and 4.3.1.1.P1.

From this observation, we hypothesized that these co-occurring AMR determinants were associated with mobile genetic elements. We utilized MOB-suite [56] to identify plasmid-associated contigs that harbour AMR genes. We detected 135 putative plasmids based on the presence of the *rep* gene across 69 genomes, with IncQ1 being the most common identified replicon type (*n*=21), followed by IncFIB (*n*=19) and IncY (*n*=17) (Fig. S6). All three replicon types have been previously implicated in the spread of AMR in *S*. Typhi [13, 78, 79] and were frequently detected in the MDR/XDR genomes of the New York State dataset. Nine AMR genes, including those conferring resistance to aminoglycosides and fluoroquinolones, were found to be associated only with the plasmid-related contigs, while five genes (*catA1*, *dfrA7*, *sul1*, *sul2* and *bla*_CTX-M-15_) were present on both chromosomal and plasmid contigs, indicating the mobility of these resistance determinants (Fig. S9). Genotypes 2.3.1, 3, 4.3.1.1 and 4.3.1.1.P1 each included genomes with contigs associated with multiple AMR genes and diverse replicon types (Fig. 3b). Isolates with genotypes 4.3.1.1 and 4.3.1.1.P1 included IncQ1-associated contigs that harboured *aph(3″) Ib*, *aph(6)-Id, bla*_TEM-1_, *catA1*, *dfrA7*, *sul1* and *sul2* genes. Additionally, we detected IncY replicon contigs that contained *aph(3″) Ib*, *aph(6)-Id, bla*_TEM-1_, *bla*_CTX-M-15_, *qnrS1* and *sul2* in genotype 4.3.1.1.P1 genomes, reflecting previous observations of XDR *S*. Typhi in Pakistan that possess an IncY plasmid conferring resistance to fluoroquinolones and third-generation cephalosporins [1, 2].

### New York State *S*. Typhi are closely related to globally dominant high-risk genotypes

**Fig. 4.**
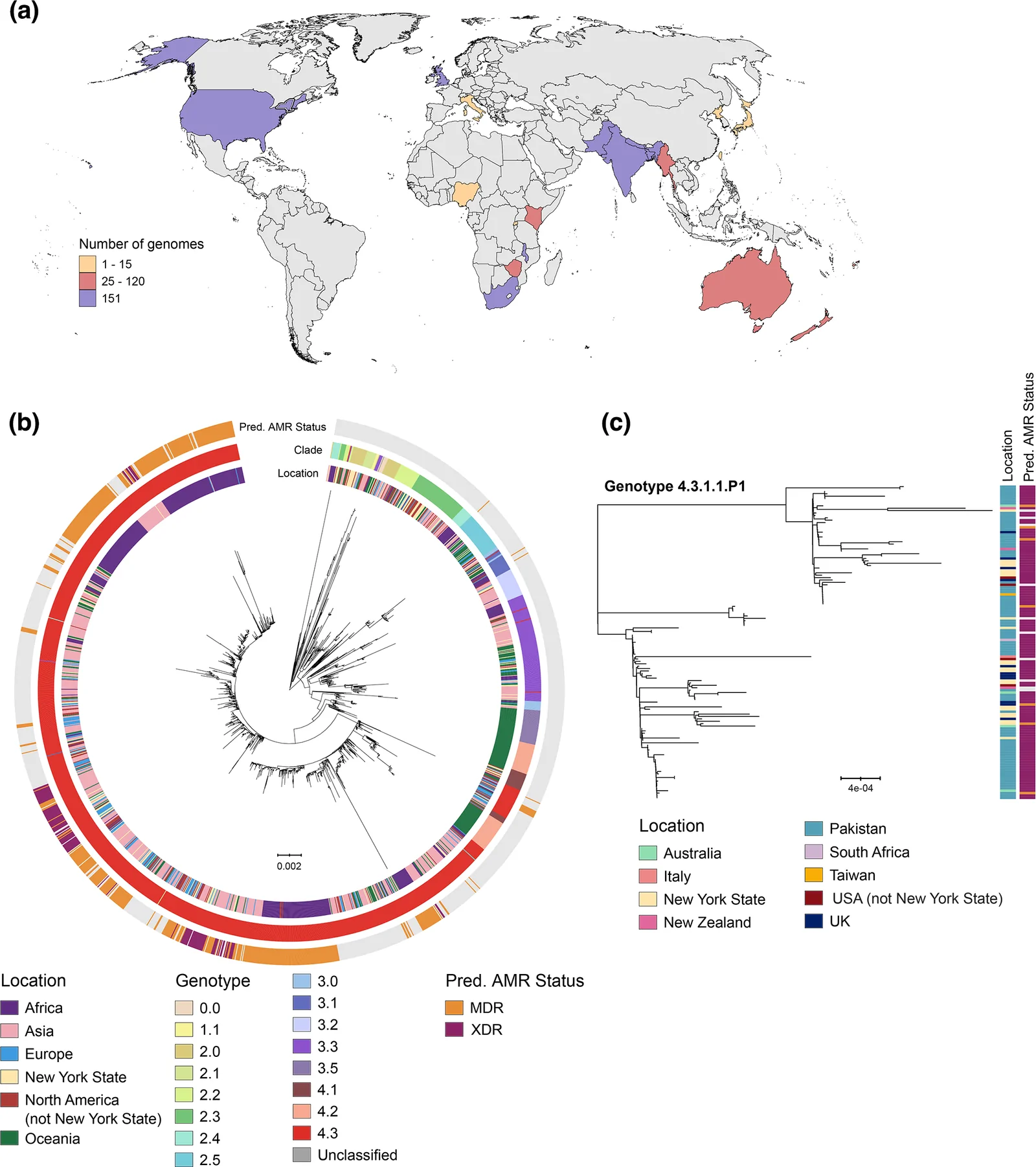
Geographical sources and phylogenetic relationship of the 151 *S*. Typhi genomes from New York State with 1,643 globally distributed genomes. (**a**) World map showing the distribution of the global dataset used in this study. Countries and New York State are coloured according to the total number of genomes included in this study. (**b**) A maximum likelihood phylogenetic tree based on a 4.16 Mbp alignment of 4,530 core genes. The tree is rooted at the midpoint. Tree scale reflects the number of SNPs/site in the core SNP alignment (37,633 SNPs). The coloured strips around the phylogeny show the location, genotype based on GenoTyphi classification [19, 20] and predicted AMR status (innermost to outermost rings). The predicted AMR status includes MDR and XDR *S*. Typhi [10, 13]. Detailed information on the genomic features, associated metadata and distribution of AMR determinants of the 1,643 global genomes is presented in Tables S2 and S7. (**c**) A close-up view of the phylogeny of XDR *S*. Typhi genotype 4.3.1.1.P1. The coloured strips around the phylogeny show the location (left coloured strip) and predicted AMR status (right coloured strip).

To place the 151 genomes from New York State in the context of a larger global *S*. Typhi population, we retrieved an additional 1,643 genomes from the TyphiNET database (Table S2) [40]. These *S*. Typhi genomes originated from 20 countries across 5 continents between 2016 and 2021, including other parts of the USA (Fig. 4a). The phylogenetic tree based on 4,530 core genes of this second dataset showed the New York State genomes intermingled with genomes from other countries and continents (Figs 4b and S3). Their phylogenetic placement indicates multiple international origins of the New York *S*. Typhi population. While we observed a few phylogenetically discordant genomes, these were rare and likely reflect biological or evolutionary processes rather than errors in phylogenetic reconstruction or genotype assignment. We further examined the 4.3.1.1.P1 genotype in the combined dataset (*n*=131 genomes) and found that nearly all of the genomes were XDR (*n*=114, 87.0%) (Fig. 4c). Additionally, while the majority of the genomes were collected from Pakistan (*n*=87, 66.4%), they intermingled with genomes from Australia (*n*=4), Italy (*n*=1), New York State (*n*=22), New Zealand (*n*=3), South Africa (*n*=1), Taiwan (*n*=1), other parts of the USA (*n*=4) and the UK (*n*=11), revealing the international spread of this endemic high-risk lineage.

**Fig. 5.**
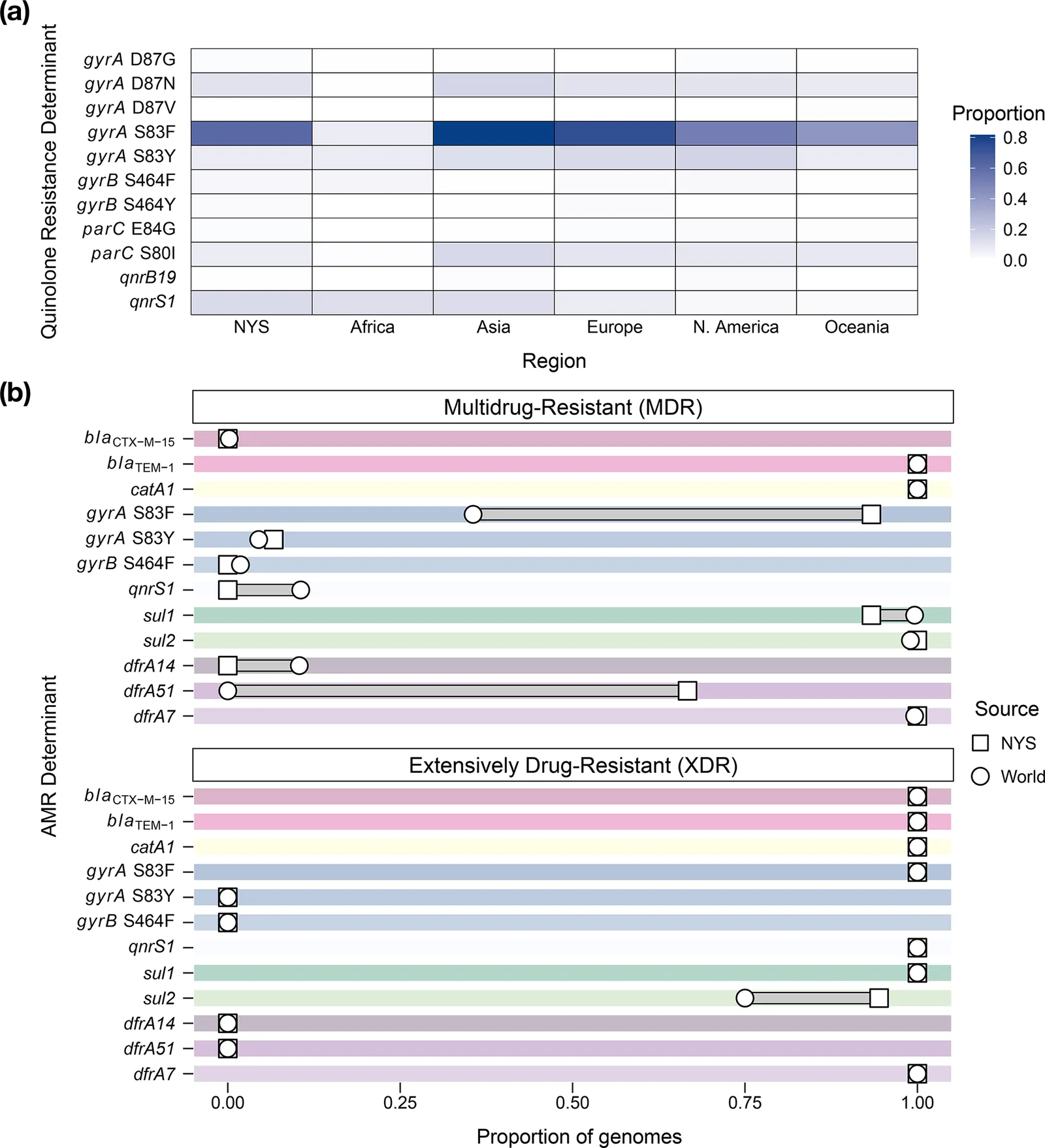
New York State and global *S*. Typhi genomes exhibit similar AMR patterns. (**a**) Heatmap showing the proportion of quinolone resistance determinants detected in pooled genomes from New York State and from each indicated continent, calculated relative to the total number of genomes in each region. North America represents the additional genomes from the USA, excluding those from New York State. (**b**) Proportion of MDR and XDR genomes harbouring the indicated AMR determinants. Squares represent the population of MDR/XDR-predicted New York State genomes (*n*=15 and 18, respectively), and circles represent the population of global MDR/XDR genomes (*n*=492 and 96, respectively). Differences in proportions between the two datasets are highlighted by grey segments.

Finally, we sought to determine if the diverse set of AMR determinants in our New York State dataset was associated with that of the international *S*. Typhi genomes. First, we analysed the broad-level geographic distribution of genetic elements associated with quinolone resistance of the New York State and global datasets. New York State *S*. Typhi exhibited similar patterns of fluoroquinolone resistance relative to Asia, Europe, North America (representing the USA but non-New York State isolates) and Oceania (Fig. 5a). The QRDR mutation *gyrA* S83F frequently detected in our local dataset was also predominant in these other locations. As expected, this mutation and other quinolone resistance determinants were largely absent from the African genomes, as XDR *S*. Typhi has not yet emerged on this continent [10]. Next, we examined the MDR and XDR genomes to identify shared AMR profiles. We detected near-universal presence of *bla*_TEM-1_, *catA1*, *sul2* and *dfrA7* across the MDR *S*. Typhi genomes (Fig. 5b). Additional trimethoprim resistance genes (*dfrA14* and *dfrA51*) and quinolone resistance determinants (*qnrS1* and *gyrA* S83F) had differences in proportion between the datasets, with *dfrA51* and *gyrA* S83F exhibiting higher prevalence in the global genomes relative to those from New York State. The XDR genomes displayed close concordance between the two datasets; the AMR profiles common across all XDR genomes included *bla*_CTX-M-15_, *bla*_TEM-1_, *catA1*, *gyrA* S83F, *qnrS1*, *sul1* and *dfrA7*. In contrast, the sulphonamide resistance gene *sul2* was more frequently observed in the XDR genomes in New York State relative to those with global origins. Taken together, these results show the international origins of the New York State MDR/XDR genomes revealed by similarities in AMR patterns with geographically diverse lineages, including those endemic in other parts of the world.

## Discussion

In this study, we analysed 151 *S*. Typhi genomes from New York State to elucidate the population structure and its pool of AMR determinants. Our multi-year statewide genomic surveillance of *S*. Typhi will be valuable in early detection of emerging MDR *S*. Typhi, monitoring of the geographical dissemination of AMR determinants and identifying shifts in regional *S*. Typhi populations, such as those caused by clonal diversification, replacement or expansion. Close interaction and cooperation between public health laboratories and genomic scientists, especially related to data sharing, remains essential in outbreak preparedness and response capabilities. Our study also underscores the need for the implementation of consistent genomic methods, from sequencing to phylogenetics and *in silico* gene screening, among public health laboratories, which will be critical to a systematic surveillance of *S*. Typhi across state lines and national borders. This is particularly important in regions where typhoid fever is not endemic and the population structure of *S*. Typhi is strongly linked to human mobility.

The New York State *S*. Typhi population consists of globally prominent genotypes that emerged in regions where typhoid fever is endemic. The phylogenetic analysis further confirmed the close evolutionary relationships of our genomes with those from other countries, thus reinforcing the importance of closely monitoring the global dissemination of high-risk *S*. Typhi genotypes. In our study, the composition of genotypes in New York State is consistent with those from other non-endemic countries that reported occasional intercontinental import via human travel followed by local clonal expansion and/or diversification. This is especially true for genotype 4.3.1, whose international dominance including its presence in non-endemic regions is shaped mainly through multiple intercontinental importation and intracontinental transmission events [15, 21, 80]. Our results in New York State add to the current knowledge about the global reach of genotype 4.3.1/H58.

Vigilance is critical to curb the emergence and evolution of genotypes that may harbour novel features that have not been encountered before. In fact, the emergence of *S*. Typhi strains with additional or novel combinations of AMR determinants acquired via mobile genetic elements has already been documented. For example, the MDR 4.3.1/H58 clone endemic in Pakistan (4.3.1.1.P1) transformed into XDR through the acquisition of a plasmid encoding resistance to fluoroquinolones and third-generation cephalosporins from an *Escherichia coli* strain or another enteric bacterial donor [13]. This new XDR variant has been circulating in Pakistan since at least 2010 but was first detected only in 2016 and eventually reached the UK via a traveller [13]. In India, a new clone of ceftriaxone-resistant *S*. Typhi (genotype 4.3.1.2.2) was identified in 2022–2023 and exhibited rapid expansion and regional domination [81]. This Indian clone harbours three plasmids, including a multidrug resistance IncFIB(K) procured from other *Enterobacterales* [81]. It may be only a matter of time before this clone reaches regions outside of India. While we do not have travel history data of patients from whom our *S*. Typhi samples were obtained, previous studies in non-typhoid-endemic regions, such as in England [82, 83], Australia [84], Italy [85] and Denmark [86], involved travellers returning from South Asia and East and West Africa where the burden of typhoid fever is high [5, 87]. More worrying is that the global risk of typhoid outbreaks caused by XDR *S*. Typhi is strongly associated with international air travel facilitating rapid importation followed by sustained onward transmission [88]. Genomic surveillance of *S*. Typhi in the USA will certainly improve with integration of travel history data of typhoid fever patients. Such information is also essential in distinguishing travel-related importation from long-term carriage, as in the three typhoid cases within a household in Queensland, Australia, whereby the index case chronically carried *S*. Typhi for at least 12 years [89]. Similarly, in 2019, typhoid fever due to an XDR strain was identified among residents in Illinois, USA, with no history of international travel and none had shared contacts nor close contact with ill persons [90].

Another important finding in our study is that New York State *S*. Typhi genomes carry a multitude of acquired genes and mutations associated with resistance to nine classes of antimicrobial agents, including predicted MDR and XDR profiles. Notable is the wide variety of point mutations (in *gyrA*, *gyrB* and/or *parC*) and genes (*qnr*) associated with quinolone resistance that were detected in the entire population, which have also been documented in *S*. Typhi elsewhere [77, 91]. This assemblage of quinolone resistance determinants shows that selection pressures imposed by antimicrobial agents help drive *S*. Typhi to develop a variety of evasion mechanisms [77, 91]. While the contribution of all the known resistance mechanisms against quinolones has not been fully investigated, their additive or synergistic interactions may impart elevated levels of quinolone resistance [77] and even a reduction in the efficacy of older fluoroquinolones [92]. Mutations in *gyrA* and *parC* conferring quinolone resistance have been shown to be associated with significant fitness benefits, even in the absence of antimicrobial exposure [93].

The variety of quinolone resistance determinants observed in the New York State population mirrored that of the multinational TyphiNet dataset, indicating that these genetic elements were already present in these lineages prior to their detection in New York State. Moreover, the observed co-occurrence of some AMR determinants is due in part to the presence of mobile genetic elements within the New York State isolates. The three-way association between the sulphonamide resistance gene *sul2* and the resistance genes *aph(3′)-Ib* and *aph(6)-Id* was unexpected, as aminoglycosides are ineffective against intracellular pathogens such as *S*. Typhi [94, 95]. However, our analysis indicates that these elements are present simultaneously on plasmid-associated contigs, which mirrors previous observations of co-acquisition of diverse AMR determinants via mobile genetic elements in *S*. Typhi and non-typhoidal *Salmonella* serovars [96–98]. We also observed an association between the fluoroquinolone resistance gene *qnrS1*, the beta-lactam resistance gene *bla*_TEM-1_ and extended spectrum beta-lactamase gene *bla*_CTX-M-15_ with IncQ, IncX and IncY plasmid replicons in our dataset, indicating that these resistance determinants were likely horizontally acquired. As with the QRDR mutations, the presence of these genes in both the New York State and global TyphiNet genomes suggests shared genetic backgrounds linked to localized reservoirs and international dissemination via travel. Acquisition of AMR determinants conferring resistance to fluoroquinolones and third-generation cephalosporins via mobile genetic elements is a concerning trend observed for H58-associated lineages of *S*. Typhi, as it enables further spread of multidrug and extensive drug resistance [13, 80, 99]. An in-depth investigation of their transfer mechanisms is therefore critical.

We acknowledge the limitations of our study. First, our genomic dataset lacks pertinent data related to the clinical, demographic, socio-economic and travel history of patients from which the *S*. Typhi isolates were sampled from. This information was not provided by healthcare providers to the Wadsworth Center during statewide surveillance. We therefore underscore the importance of integrating patient information with molecular data in epidemiological studies to precisely trace person-to-person transmission patterns, ascertain origins of resistant genotypes and uncover disease outbreaks, thereby guiding public health interventions that are most effective at local or regional scales. In our study, the detection of geographically localized genotypes (e.g., Pakistan-associated genotype 4.3.1.1.P1 and Bangladesh-associated genotype 3.3.2.Bd1) indicates potential transmission events that may have been facilitated by long-distance travel from these endemic regions; however, we can only speculate on how they were introduced to New York State in the absence of patient-associated information. Second, the isolates included in this study were those from patients clinically diagnosed by their healthcare providers. Hence, they do not encompass the actual *S*. Typhi population present in the state, with *S*. Typhi infections that are undetected, unconfirmed or unreported remaining invisible to the current surveillance programme. Asymptomatic carriers of *S*. Typhi are particularly important in epidemiological studies, especially in making accurate inferences about the direction of pathogen transmission [7, 100], but our study does not include samples from these individuals. Third, predicted AMR profiles in our study were not validated by phenotypic antimicrobial sensitivity testing, which also impeded our ability to analyse genotype–phenotype correspondence. The presence of mutations and acquired genes alone does not confirm their phenotypic features. However, we note that previous studies that carried out comparisons of phenotypic and genome-based AMR determination in *S*. Typhi and *S*. Paratyphi demonstrated 99.97–100% concordance; thus, sequence data is a robust and useful approach to infer AMR characteristics in enteric fever strains [83, 101]. Lastly, our use of short-read sequences means that we cannot resolve complete plasmid sequences and their cargo genes. Notwithstanding these limitations, our study presents an informative genome-based view of the standing genetic variation and AMR determinants present in New York State where typhoid fever is not endemic.

In summary, our findings reveal the presence of globally dominant genotypes of *S*. Typhi carrying multiple AMR determinants in New York State. These findings expand existing knowledge about the diversity and genomic features of *S*. Typhi in non-endemic regions, which are critical to implementing efforts to minimize secondary transmission and public health control measures against *S*. Typhi that are most effective at local and regional scales.

## Supplementary material

10.1099/mgen.0.001816 Supplementary Material 1.

10.1099/mgen.0.001816 Supplementary Material 2.

## Abbreviations

AMR: antimicrobial resistance
BHI: Brain Heart Infusion
CDC: Centers for Disease Control and Prevention
H58: haplotype 58
MDR: multidrug resistant
NCBI: National Center for Biotechnology Information
QRDR: quinolone resistance-determining region
SRA: Sequence Read Archive
*S*. Typhi: *Salmonella enterica* subspecies *enterica* serovar Typhi
TSI: triple sugar iron
XDR: extensively drug resistant
