# Supplementary material for: Presence of globally prominent multidrug-resistant genotypes of Salmonella enterica serovar Typhi in New York State, 2016–2023
Source: Microb Genom. 2026 Sep 2;12(9):001816. doi: 10.1099/mgen.0.001816 (PMC13537747; doi:10.1099/mgen.0.001816)
Supplement: Supplementary file 1 [file mgen-12-01816-s001.pdf]

Presence of globally prominent multidrug-resistant genotypes of *Salmonella enterica* serovar Typhi in New York State, 2016 – 2023

Samantha K. Lindberg[1], Ana Beatriz Garcez Buiatte[1], Odion O. Ikhimiukor[2], Leticia Roberta Martins Costa[1], Samantha E. Wirth[2], Kimberlee A. Musser[2], Lisa A. Mingle[2], Cheryl P. Andam[1#]

[1] Department of Biological Sciences, University at Albany, State University of New York, Albany, New York, USA
[2] Wadsworth Center, New York State Department of Health, Albany, New York, USA

Supplementary Figures

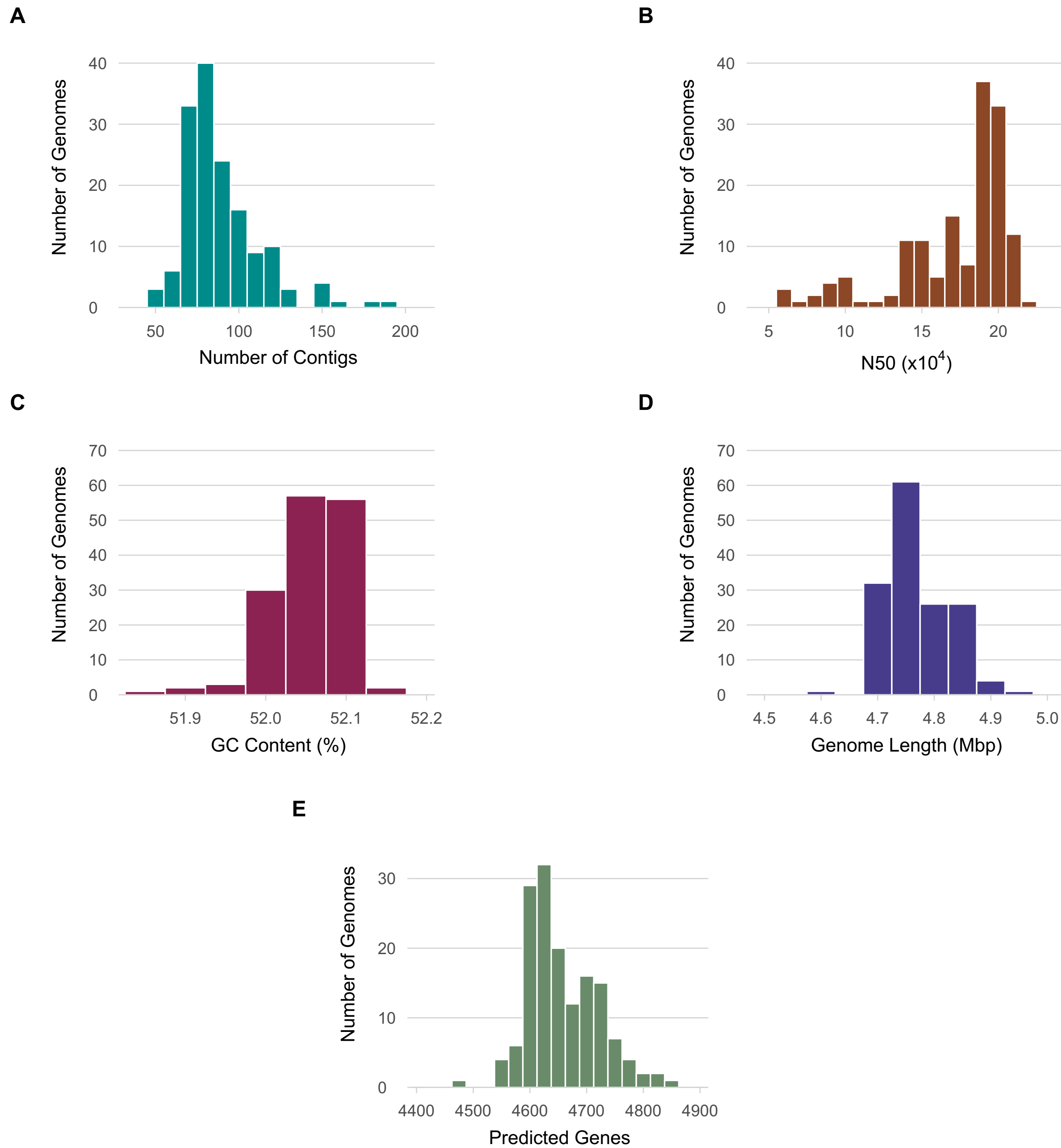

Figure S1. Quality metrics and genomic features of the 151 *S.* Typhi genomes included in this study. Histograms show the distribution of (A) the number of contigs per genome, (B) N50 per genome, (C) GC content per genome, (D) genome length per genome (Mbp), and (E) the number of predicted genes per genome. We observed a positive relationship between genome size and total plasmid nucleotides ($R^2 = 0.67$) for the New York State *S.* Typhi genomes. Detailed information for each genome is presented in Supplementary Table S1.

A

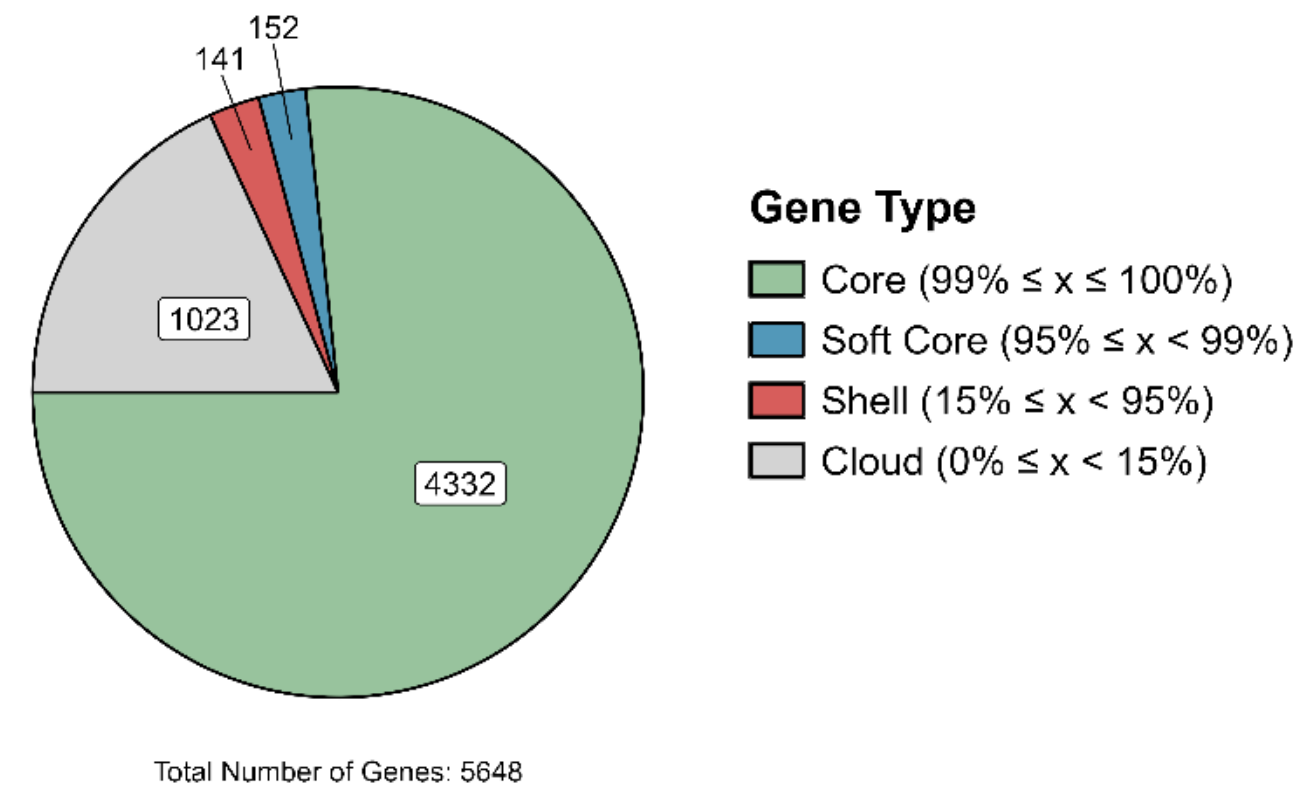

B

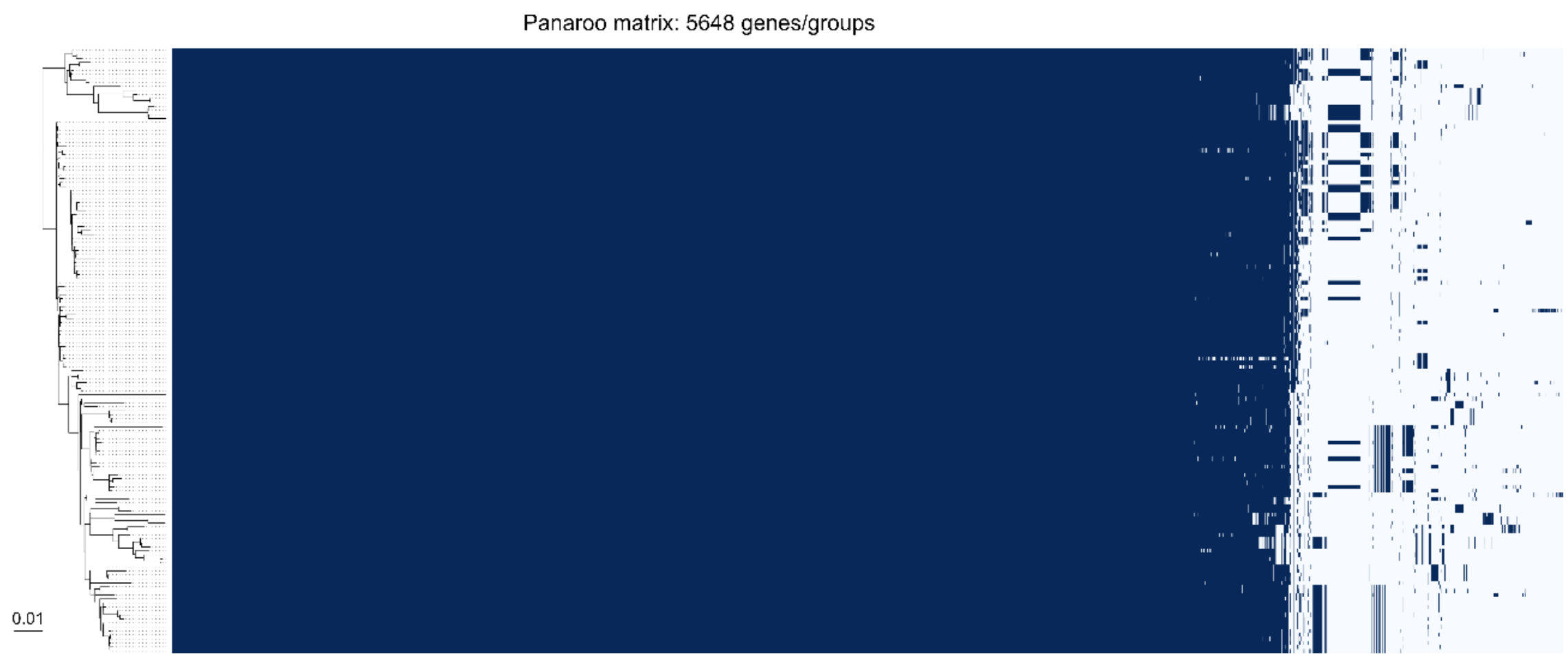

Figure S2. Components of the pan-genome of the 151 *S.* Typhi genomes determined using Panaroo. (A) Number of core, softcore, shell, and cloud genes. (B) Phylogenetic tree with gene presence-absence matrix.

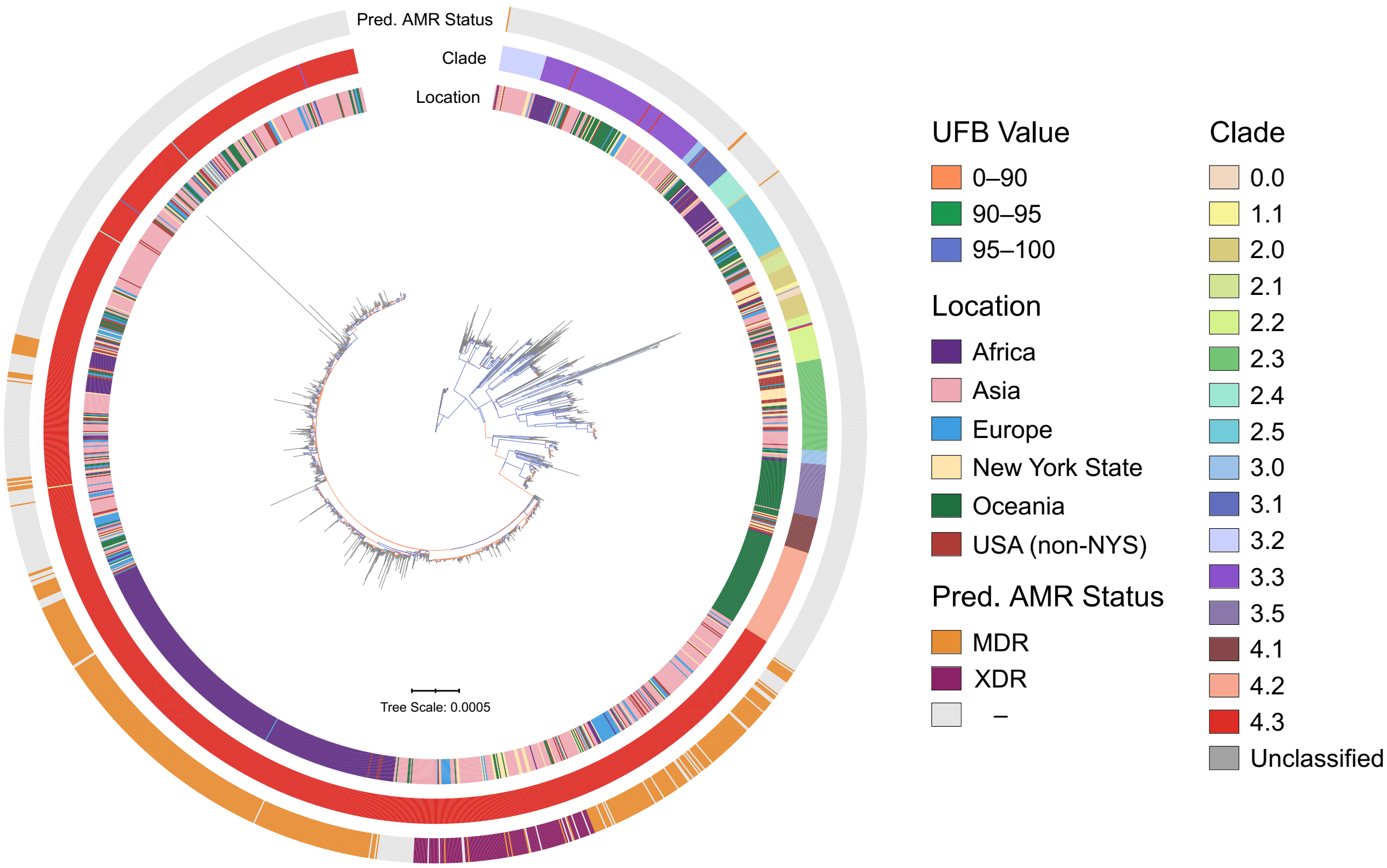

Figure S3: Phylogeny of 1,794 global *S.* Typhi genomes. Recombination-free maximum likelihood tree showing the phylogenetic relationships of the global *S.* Typhi dataset mapped to a (GenBank accession: AL513382) reference genome of *S.* Typhi CT18. Tree branches are shaded according to Ultrafast Bootstrap (UFB) values. Outer rings indicate the geographical location (inner ring), Genotyphi clade (middle ring), and predicted antimicrobial resistance status (outer ring) for each genome.

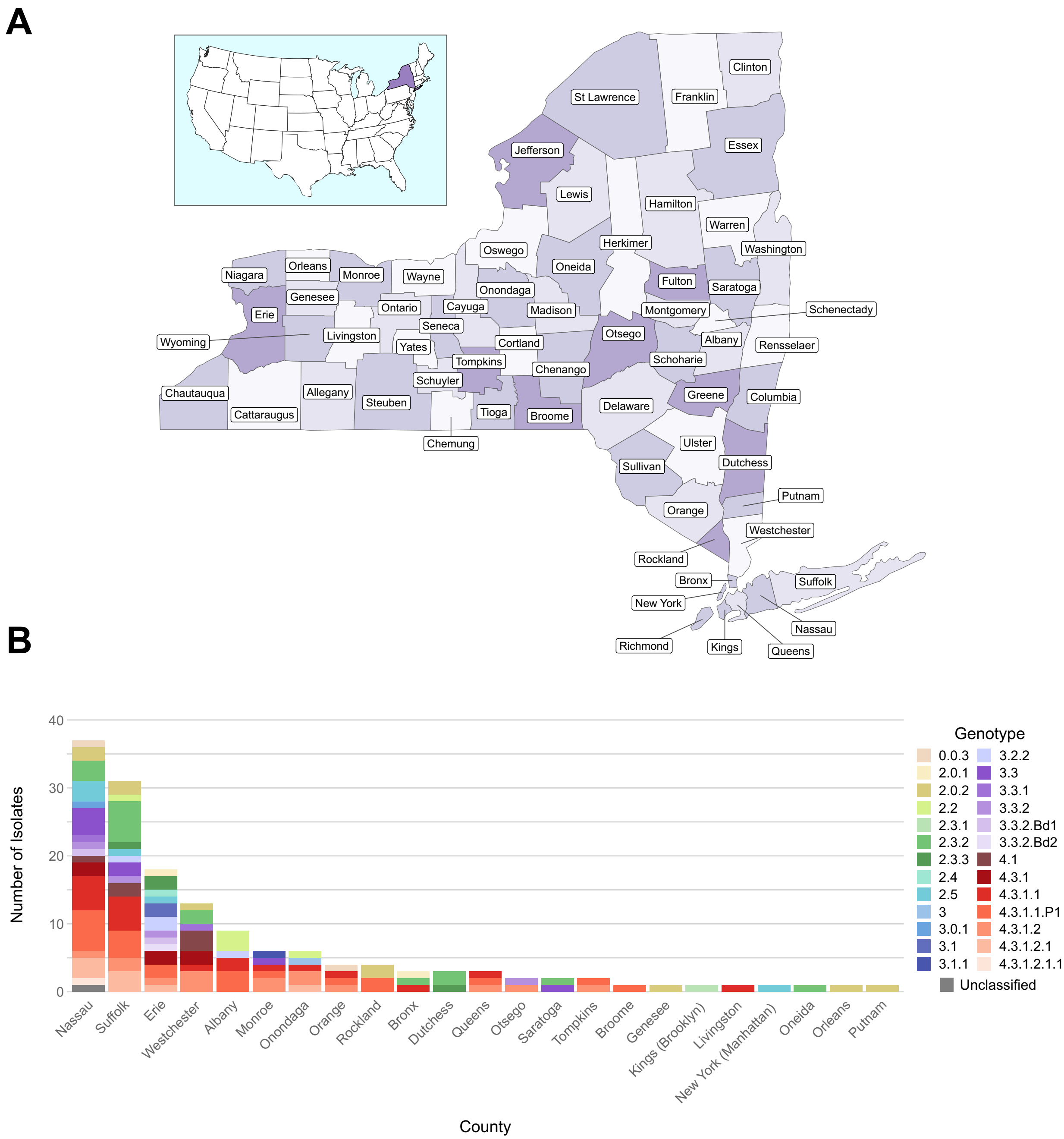

Figure S4. Detection of the 151 *S.* Typhi genomes in New York State. (A) County map of New York State. Different colors represent the boundaries of individual counties. (Inset) Location of New York State in the United States. (B) Number of *S.* Typhi genomes per county stratified by genotype.

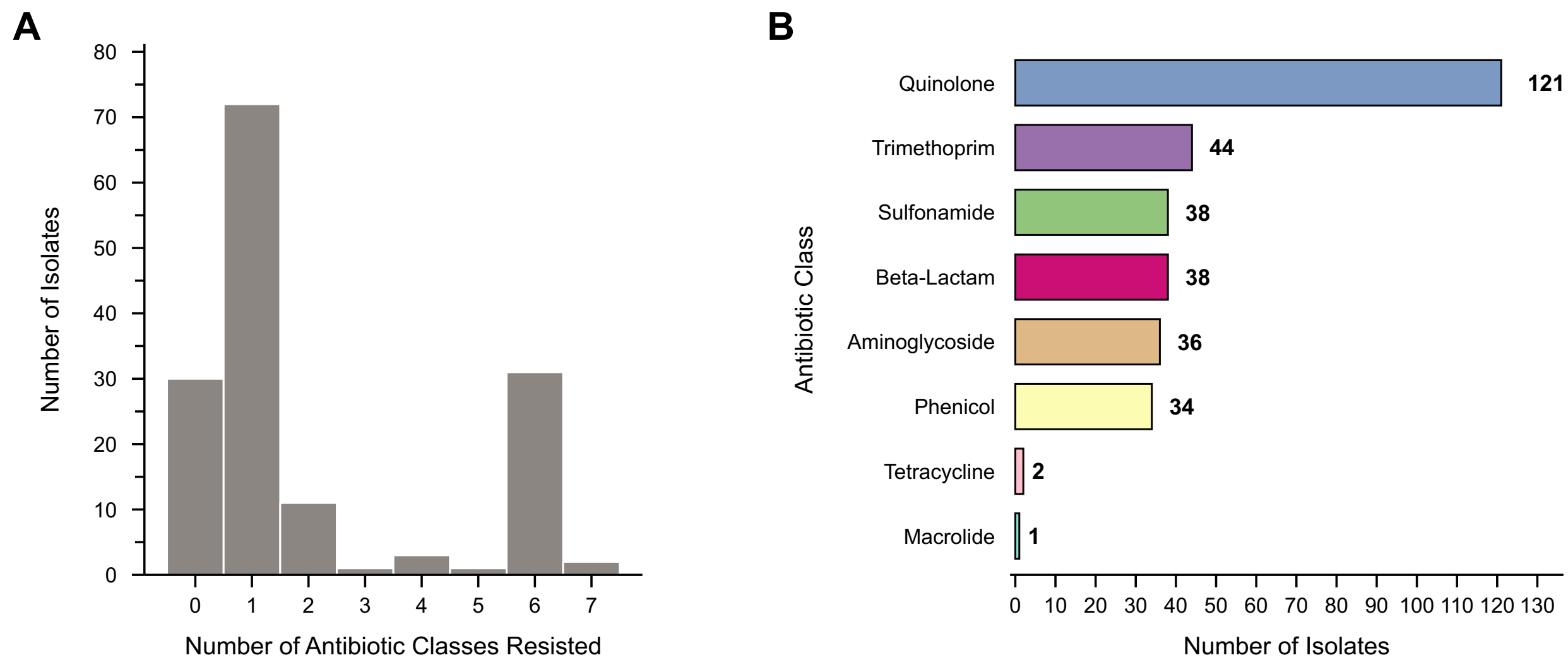

Figure S5. Antimicrobial resistance (AMR) profiles of the 151 *S.* Typhi genomes from New York State based on the presence of AMR genes and mutations. (A) Histogram of AMR determinants per genome. (B) Total number of isolates resistant to each antibiotic class.

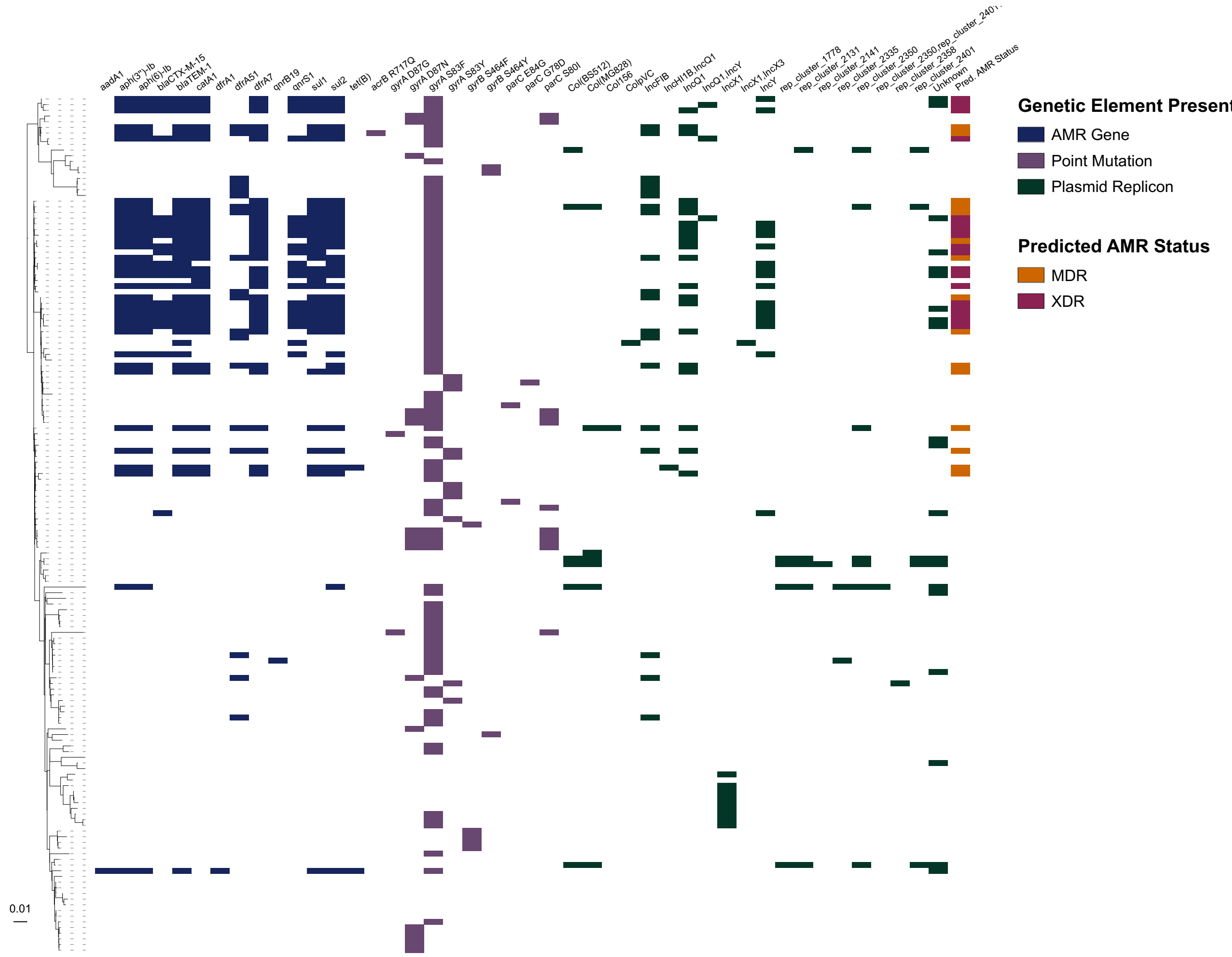

Figure S6. Distribution of antimicrobial resistance (AMR) determinants and plasmid replicon types in 151 *S.* Typhi genomes in New York State. The midpoint-rooted maximum likelihood phylogenetic tree is identical to that in Figure 1C. The matrix shows that presence or absence of specific AMR determinants (genes and mutations) and plasmid replicon types in each genome. Predicted AMR status (MDR = multidrug resistant; XDR = extensively drug resistant) were determined based on specific combinations of resistance markers based on references (Klemm et al., 2018; Marchello et al., 2020): chloramphenicol + ampicillin + trimethoprim-sulfamethoxazole for MDR, and MDR + fluoroquinolone + third-generation cephalosporin for XDR. Colored blocks represent the presence of the genetic element.

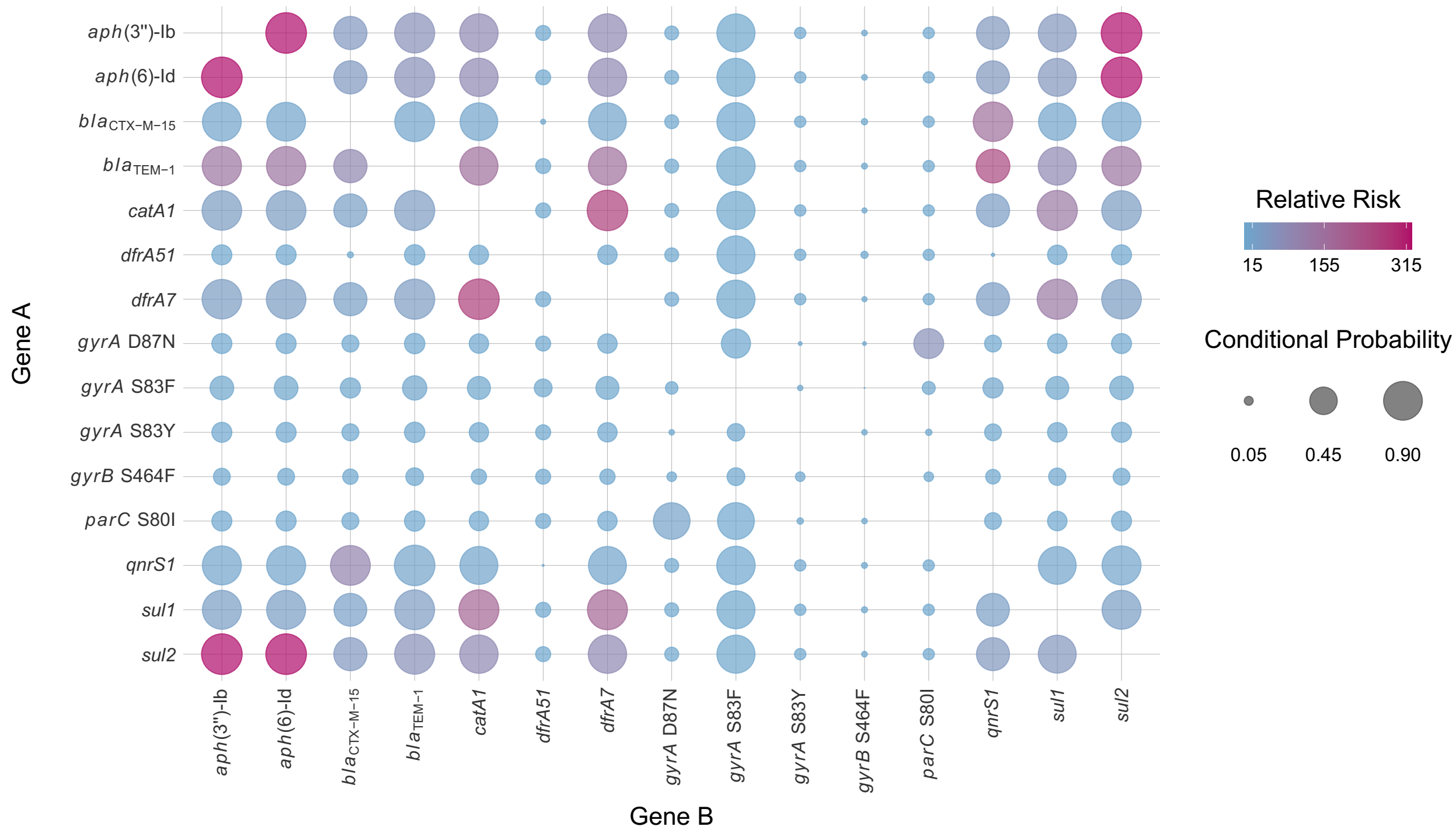

Figure S7. Co-association metrics for antimicrobial resistance (AMR) determinants detected in the 151 *S.* Typhi genomes in New York State. Bubble size is scaled by conditional probability, which represents the likelihood of observing 'Gene A' in the presence of 'Gene B'. Bubble color is scaled by relative risk, which represents the ratio of conditional probability to the probability of observing 'Gene A' in the absence of 'Gene B'. The two metrics were calculated using ReGAIN (Horvath et al., 2024).

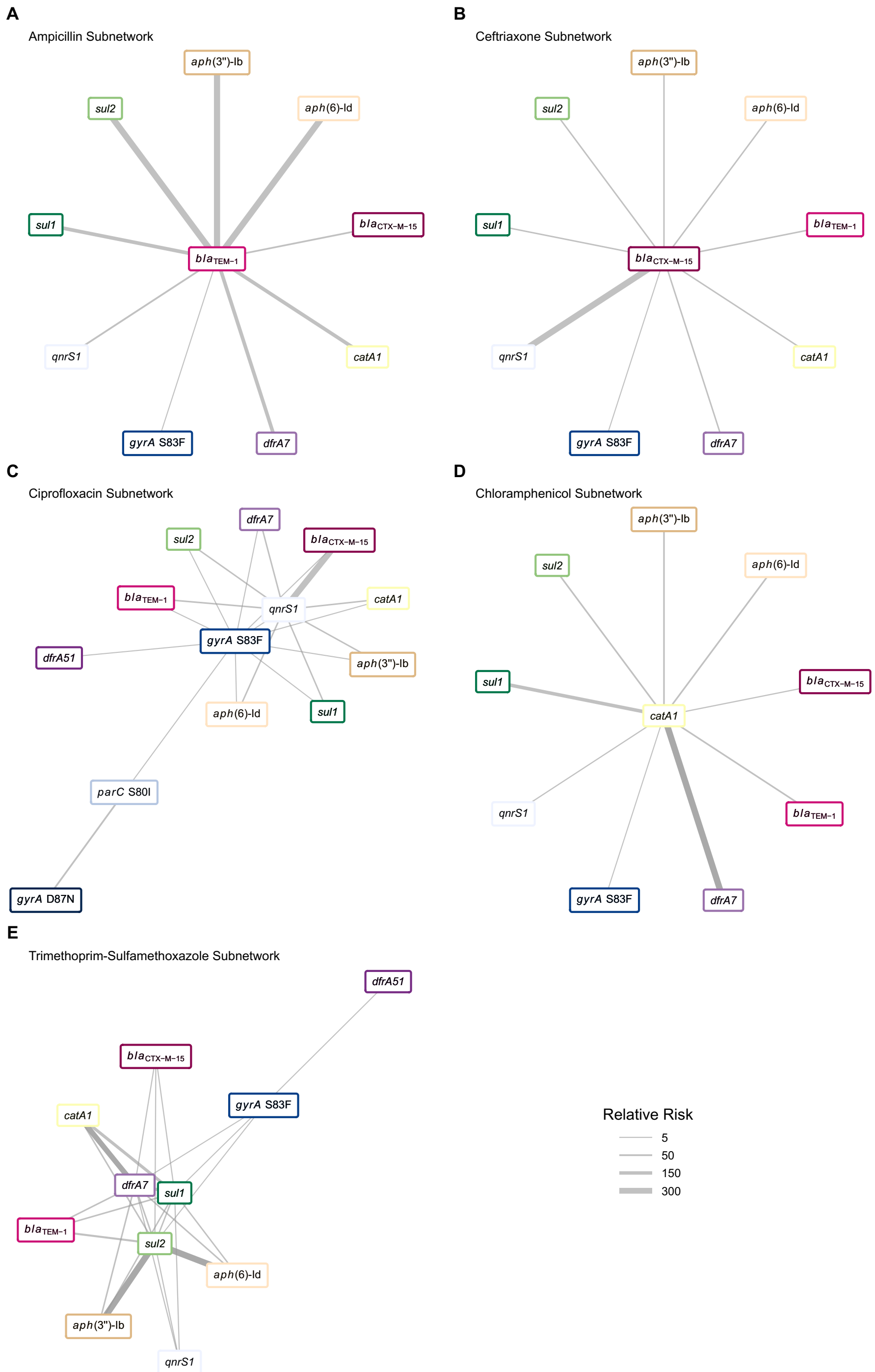

Figure S8: Co-association networks of individual AMR determinants in the 151 *S.* Typhi genomes in New York State. (A) *bla*$_{TEM\text{-}1}$ encoding ampicillin resistance, (B) *bla*$_{CTX-M-15}$ encoding ceftriaxone resistance, (C) *gyrA* S83F and *qnsR1* encoding ciprofloxacin resistance, (D) *catA1* encoding chloramphenicol, and (E) *dfrA* and *sul* encoding trimethoprim-sulfamethoxazole resistance. Edge width is scaled by relative risk, which represents the ratio of probability of observing ‘Gene A’ in the presence of ‘Gene B’ to the probability of observing ‘Gene A’ in the absence of ‘Gene B’. Relative risk values and networks were determined using ReGAIN (Horvath et al., 2024).

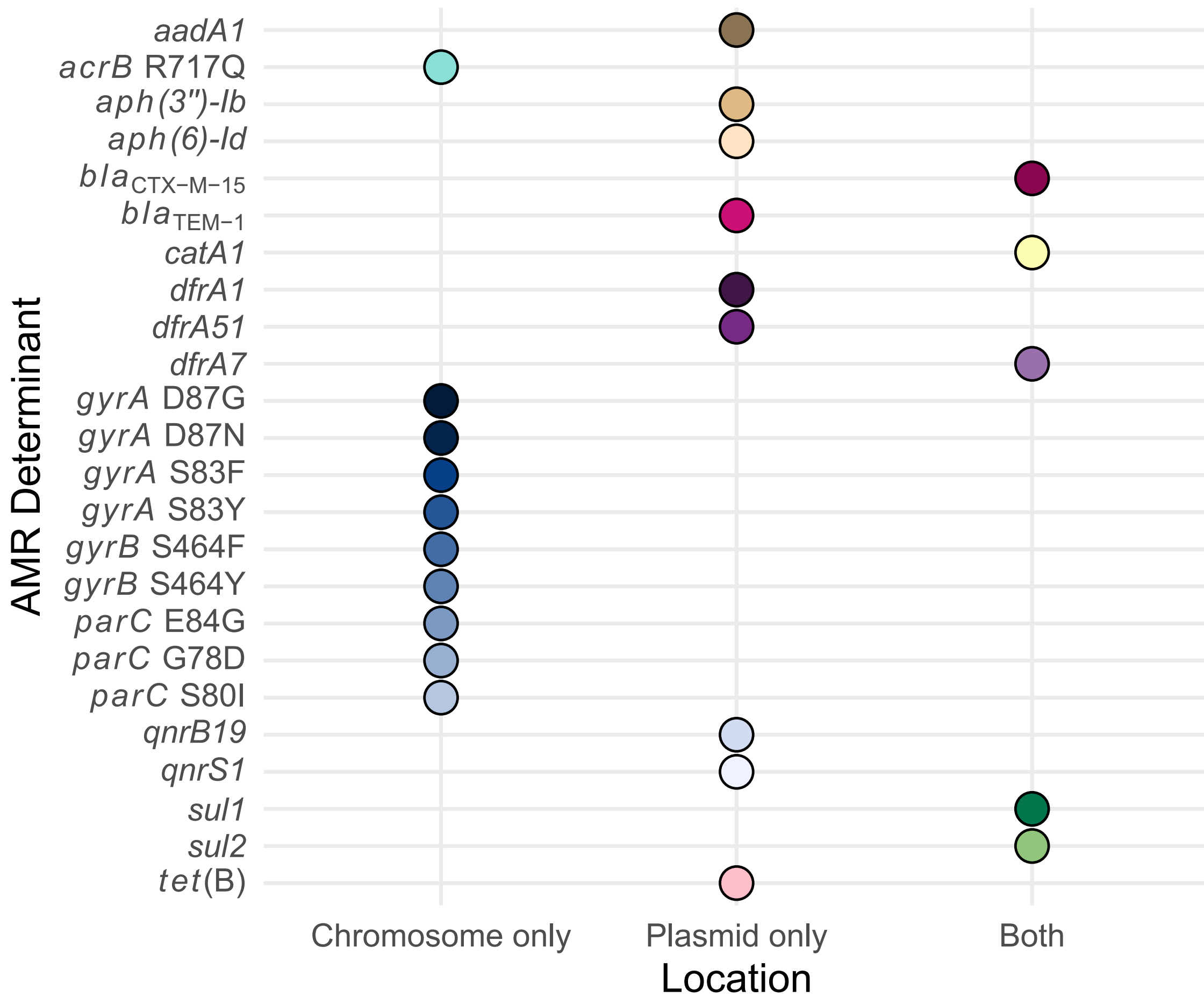

Figure S9: Distribution of AMR determinants detected on chromosome-associated contigs, plasmid-associated contigs, or both. Each filled circle indicates the genomic location of the corresponding AMR determinant.
